# Ligand-activated PPARγ downregulates CXCR4 gene expression through a novel identified PPAR response element and inhibits breast cancer progression

**DOI:** 10.18632/oncotarget.11371

**Published:** 2016-08-18

**Authors:** Daniela Rovito, Giulia Gionfriddo, Ines Barone, Cinzia Giordano, Fedora Grande, Francesca De Amicis, Marilena Lanzino, Stefania Catalano, Sebastiano Andò, Daniela Bonofiglio

**Affiliations:** ^1^ Department of Pharmacy, Health and Nutritional Sciences, University of Calabria, Rende (CS), Italy; ^2^ Centro Sanitario, University of Calabria, Rende (CS), Italy

**Keywords:** PPARγ, CXCR4, breast cancer, CAF, PPRE

## Abstract

Stromal Derived Factor-1α (SDF-1α) and its cognate receptor CXCR4 play a key role in mediating breast cancer cell invasion and metastasis. Therefore, drugs able to inhibit CXCR4 activation may add critical tools to reduce tumor progression, especially in the most aggressive form of the breast cancer disease. Peroxisome Proliferator-Activated Receptor (PPAR) γ, a member of the nuclear receptor superfamily, has been found to downregulate CXCR4 gene expression in different cancer cells, however the molecular mechanism underlying this effect is not fully understood. Here, we identified a novel PPARγ-mediated mechanism that negatively regulates CXCR4 expression in both epithelial and stromal breast cancer cells. We found that ligand-activated PPARγ downregulated CXCR4 transcriptional activity through the recruitment of the silencing mediator of retinoid and thyroid hormone receptor (SMRT) corepressor onto a newly identified PPAR response element (PPRE) within the CXCR4 promoter in breast cancer cell lines. As a consequence, the PPARγ agonist rosiglitazone (BRL) significantly inhibited cell migration and invasion and this effect was PPARγ-mediated, since it was reversed in the presence of the PPARγ antagonist GW9662. According to the ability of cancer-associated fibroblasts (CAFs), the most abundant component of breast cancer stroma, to secrete high levels of SDF-1α, BRL reduced migratory promoting activities induced by conditioned media (CM) derived from CAFs and affected CXCR4 downstream signaling pathways activated by CAF-CM. In addition, CAFs exposed to BRL showed a decreased expression of CXCR4, a reduced motility and invasion along with a phenotype characterized by an altered morphology. Collectively, our findings provide novel insights into the role of PPARγ in inhibiting breast cancer progression and further highlight the utility of PPARγ ligands for future therapies aimed at targeting both cancer and surrounding stromal cells in breast cancer patients.

## INTRODUCTION

Breast cancer is the most common malignancy and the leading cause of cancer death in females worldwide [1]. Despite advances in prevention, early detection, and treatment, development of metastatic breast cancer is responsible for the majority of cancer-related deaths [2]. Due to the inability to accurately predict the risk of metastasis, most of patients receive adjuvant chemotherapy when diagnosed with breast cancer, but approximately 30% of these patients still relapse and die of metastatic disease within five years [3].

Several molecules such as cytokines, chemokines, growth factors and their respective receptors are involved in promoting invasion and metastasis in breast cancer. Particularly, chemokine receptors may potentially facilitate tumor dissemination at each of the key steps of metastasis, including adherence of tumor cells to endothelium, extravasation from blood vessels, metastatic colonization, angiogenesis, proliferation, and protection from the host response via activation of key survival pathways such as ERK/MAPK, PI-3K/Akt/mTOR, or Jak/STAT [4–8]. Among the cytokine receptor systems, CXCR4, a 7-transmembrane G-protein-coupled receptor for the chemokine ligand CXCL12 (formerly known as Stromal-cell Derived Factor-1alpha, SDF-1α), has been shown to be consistently expressed in human breast cancer cells, and activation of SDF-1α/CXCR4 axis is supposed to be crucial in breast cancer migration and metastasis [9–13]. Recently, it has been reported that CXCR4 is not only associated with the metastatic spread of breast cancer cells to secondary organs, but it also crucial in the dissemination from the primary tumor site [14]. It is increasingly recognized that the chemokines axis plays an important role in facilitating communication between cancer cells and non-neoplastic epithelial cells in the tumor microenvironment, which includes surrounding blood vessels, immune cells, bone marrow-derived inflammatory cells, lymphocytes and fibroblasts [15–18]. Particularly, CAFs (Cancer-Associated Fibroblasts), the most abundant cell type in breast cancer stroma, produce a plethora of growth factors, extracellular matrix proteins and chemokines, among which SDF-1α, *via* its cognate receptor CXCR4, acts through autocrine- and paracrine-signaling mechanisms to support tumor progression [19–22]. Thus, tumor stroma-directed therapies targeting CXCR4 axis that mediates this crosstalk within tumor microenvironment have recently attracted increased attention from researchers.

Peroxisome Proliferator-Activated Receptor gamma (PPARγ), a ligand-activated transcription factor belonging to the nuclear hormone receptor superfamily, apart from the well-established adipogenic and metabolic actions [23–24], has evolved to a breast cancer tumor suppressor [25–29]. Among the synthetic compounds that selectively activate PPARγ, the thiazolidinediones (TDZ), the most potent insulin-sensitizing drugs available in clinical settings [30–32], have been shown to inhibit cell proliferation and induce apoptosis in different *in vitro* and *in vivo* models of breast cancers [33–39]. Recently, it has been reported that activated PPARγ is able to reduce invasion and motility through CXCR4 downregulation in colon, lung and prostate cancer cells [40–42]. However, despite these studies, either the regulatory mechanism by which PPARγ may regulate CXCR4 expression in breast cancer cells or how PPARγ works in the context of breast tumor microenvironment remain largely unknown. Here, we have identified, for the first time, a functional PPAR responsive element within the CXCR4 promoter that is responsible of the PPARγ-mediated inhibition of CXCR4 expression in breast cancer cells. We have then shown the ability of ligand-activated PPARγ to counteract stroma-induced breast cancer cell migration and invasiveness. Finally, we have demonstrated the inhibitory effects of activated PPARγ on CXCR4 expression and migratory abilities also in CAFs as an additional mechanism that may impact breast cancer progression.

## RESULTS

### Ligand-activated PPARγ downregulates CXCR4 expression and its gene promoter activity in breast cancer cells

**Figure 1:**
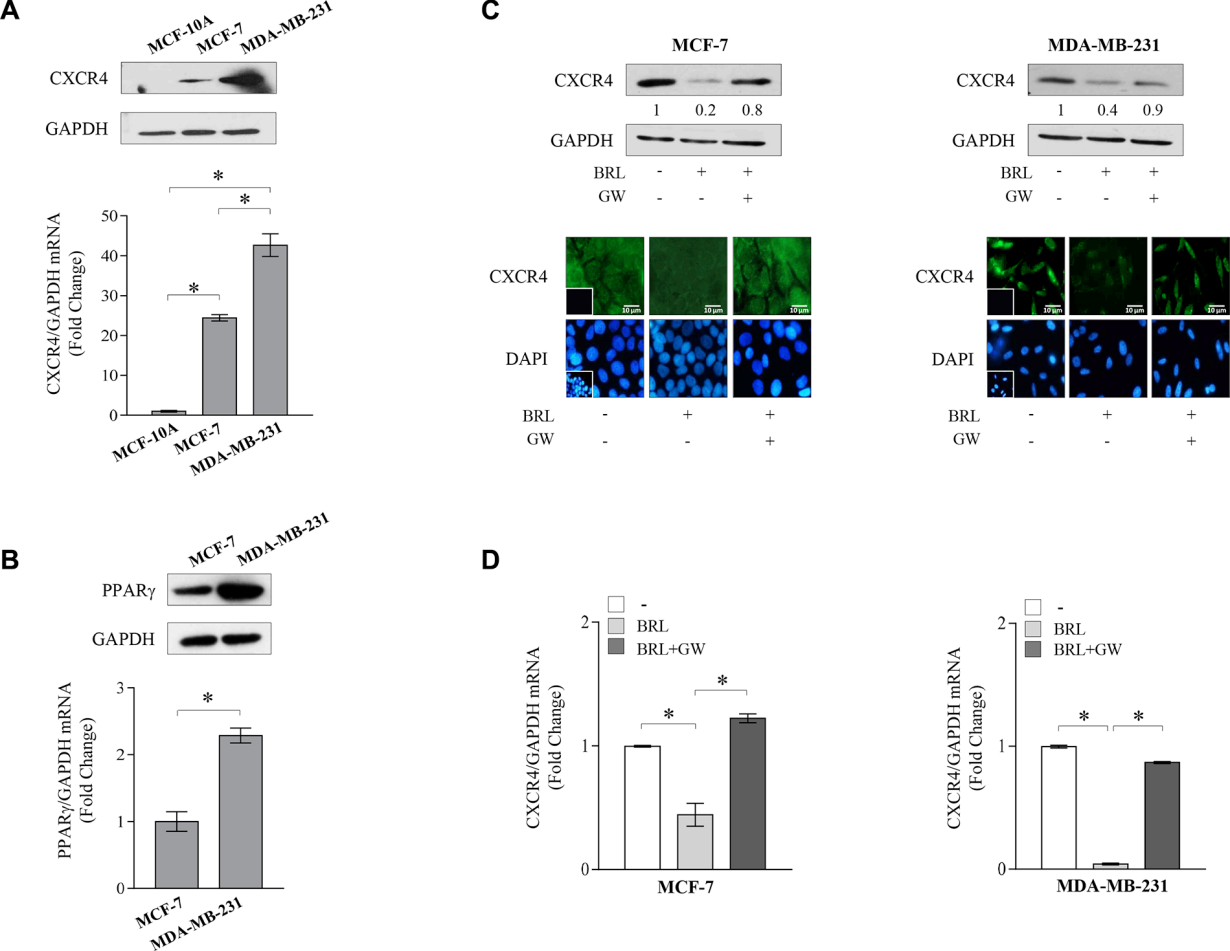
Ligand-activated PPARγ downregulates CXCR4 expression in breast cancer cells (**A**) Immunoblots (*upper panel*) and real-time RT-PCR (*lower panel*) of CXCR4 expression in MCF-10A non tumorigenic breast epithelial cells, MCF-7 and MDA-MB-231 breast cancer cells. GAPDH was used as loading control. Each sample was normalized on its GAPDH mRNA content. The results are expressed as fold change compared to breast epithelial cells. (**B**) Immunoblots (*upper panel*) and real-time RT-PCR (*lower panel*) of PPARγ expression in MCF-7 and MDA-MB-231 breast cancer cells. GAPDH was used as loading control. Each sample was normalized on its GAPDH mRNA content. The results are expressed as fold change compared to MCF7 cells. (**C**) Immunoblots (*upper panels*) and immunofluorescence (*middle panels*) of CXCR4 protein expression in MCF-7 and MDA-MB-231 cells treated with vehicle (−), BRL 10 *μ*M with or without GW 10 μM for 24 h. GAPDH was used as loading control. Numbers below the blots represent the average fold change between CXCR4 and GAPDH protein expression *vs* vehicle-treated cells. 4,6-Diamidino-2-phenylindole (DAPI) was used for the determination of the nuclei. Small squares, negative controls. Scale bar, 10 μm. (**D**) Real-time RT-PCR of CXCR4 expression in MCF-7 and MDA-MB-231 cells treated with vehicle (−), BRL 10 *μ*M with or without GW 10 μM for 12 h. Each sample was normalized on its GAPDH mRNA content. The results are expressed as fold change compared to vehicle-treated cells. The values represent the mean ± SD of three different experiments, each performed with triplicate samples. *P < 0.05. GAPDH, glyceraldehyde-3-phosphate dehydrogenase.

Previous evidences have indicated that tumor cells express distinct, tumor type-specific, nonrandom patterns of chemokine receptors and that signaling through these receptors is crucial for chemotactic migration, invasion and cancer metastasis [43–44]. CXCR4 is one of the most common chemokine receptor that has been demonstrated to be over expressed in human cancers, while its expression is low or absent in many normal tissues, including breast [14], emphasizing a critical role for this chemokine receptor in modulating cancer cell behavior. Thus, we first aimed to evaluate protein and mRNA expression levels of CXCR4 in non-tumorigenic breast epithelial cells, MCF-10A, and in two different human breast cancer cell lines by immunoblotting and qRT-PCR analyses. As shown in Figure 1A, CXCR4 expression was detected at very low levels in MCF-10A cells in respect with ERα-positive MCF-7 breast cancer cells, while higher CXCR4 levels were observed in ER-negative MDA-MB-231 breast cancer cells, which are well-characterized in terms of their metastatic potential and properties. Rosiglitazone (BRL), a PPARγ agonist used in type 2 diabetes treatment, has been shown to inhibit CXCR4 expression and to reduce the malignancy in colon, lung and prostate cancer cells [40–42]. Therefore, we evaluated PPARγ expression in MCF-7 and MDA-MB-231 breast cancer cells (Figure 1B) and assessed the effects of BRL on CXCR4 expression at both protein and mRNA levels in both cell lines. We found that BRL at 10 μM significantly reduced CXCR4 expression as evaluated by immunoblotting as well as immunofluorescence (Figure 1C) and qRT-PCR (Figure 1D) analyses in both cells. Treatment with the natural PPARγ ligand 15-Deoxy-delta12,14-prostaglandin J2 (PGJ2) at 10 μM also significantly reduced CXCR4 expression in MCF-7 and MDA-MB-231 cells (Supplementary Figure S1A). To investigate the direct involvement of PPARγ in the downregulation of CXCR4 induced by BRL, cells were treated with the PPARγ antagonist, GW9662 (GW). We found that the reduction of CXCR4 levels induced by PPARγ ligands was completely abrogated in the presence of GW treatment (Figure 1C and 1D, Supplementary Figure S1A), addressing that these effects on CXCR4 expression were mediated by PPARγ. Using siRNA technology, we confirmed the specific role of PPARγ in regulating CXCR4 expression in both cell lines (Supplementary Figure S1B).

### Identification of a functional PPAR responsive element (PPRE) within the CXCR4 promoter

**Figure 2:**
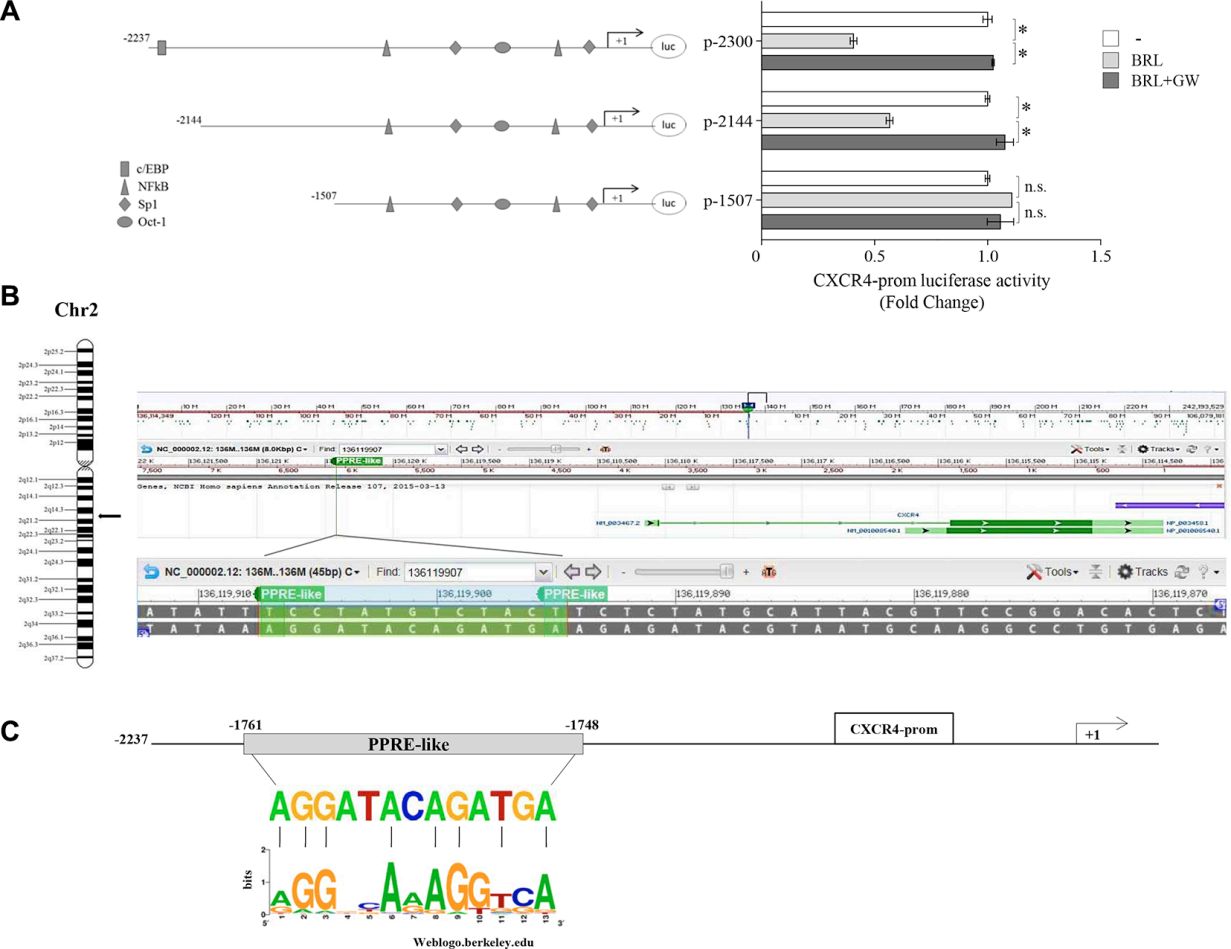
PPARγ modulates the transcriptional activity of CXCR4 gene promoter containing a putative PPAR response element (PPRE) (**A**) Schematic representation of the CXCR4 promoter constructs used in this study (*left panel*). MCF-7 cells were transiently transfected with luciferase plasmids containing the CXCR4 promoter (p-2300) and its deleted constructs (p-2144 and p-1507) and then treated with vehicle (−), BRL 10 μM with or without GW 10 μM for 12 h (*right panel*). The results are expressed as fold change respect to the vehicle-treated cells (−). The results are mean ± SD of three different experiments, each performed with triplicate samples. *P < 0.05. n.s. = not significant. (**B**) Chromosomal localization of the human *cxcr4* gene at chromosome 2 (*left panel*). A shot from NCBI genome browser to illustrate the localization of *cxcr4* gene. The location of Peroxisome proliferator response element (PPRE)-like is highlighted by vertical line and zoomed-in to view the genomic sequence spanning from 136119907 to 136119895 base pair in the negative strand (*right panel*) (**C**) The genomic sequence of the PPRE-like motif within CXCR4 promoter is aligned to a logo graphic representation of PPRE sequence generated using a PPRE collection with WebLogo [48].

**Figure 3:**
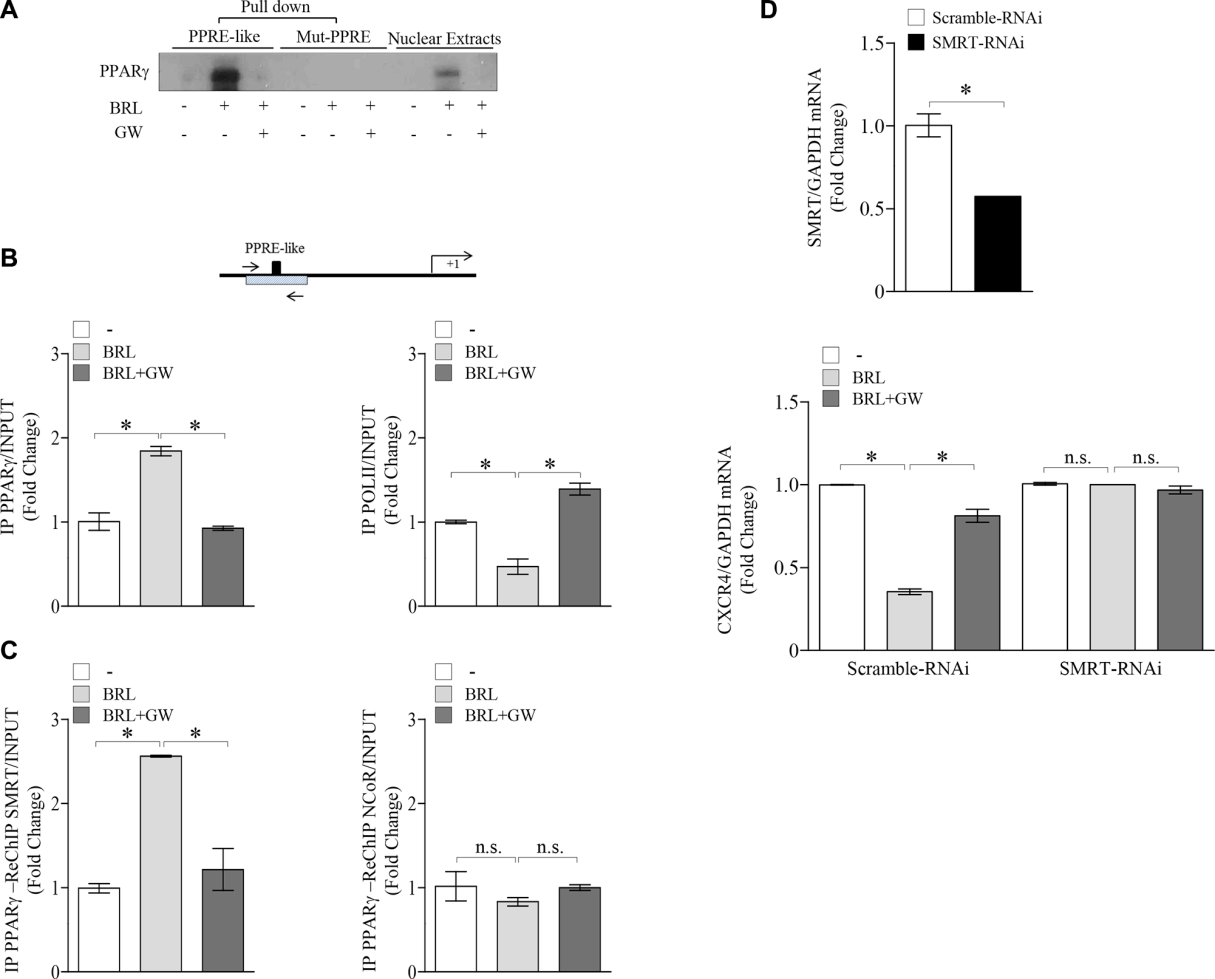
Ligand-activated PPARγ binds to a PPRE-like site within CXCR4 promoter (**A**) DAPA on nuclear extracts from MCF-7 cells treated with vehicle (−), BRL 10 *μ*M with or without GW 10 μM for 3 h. PPRE-like or mutated (Mut-PPRE) biotinylated oligonucleotides were used. Nuclear Extracts, positive control. (**B**) Schematic representation (*upper panel*) of PPRE-like site in CXCR4 promoter region. Chromatin Immunoprecipitation (ChIP) assay (*lower panel*) with anti-PPAR*γ* and anti-POL II antibodies in MCF-7 cells treated with vehicle (−), BRL 10 μM with or without GW 10 μM for 1 h. (**C**) ChIP with the anti-PPAR*γ* antibody was re-immunoprecipitated (Re-ChIP) with the anti-SMRT or anti-NCOR antibodies. The CXCR4 promoter sequence including the putative PPRE site was detected by Real-time-PCR with specific primers (see Material and Method section). (**D**) mRNA levels of SMRT (*upper panel*) and CXCR4 (*lower panel*) evaluated by Real-time RT-PCR in MCF-7 cells transfected with control RNAi (Scramble RNAi) or SMRT RNAi for 24 h and then treated with vehicle (−), BRL 10 *μ*M with or without GW 10 μM for 24 h as indicated. Each sample was normalized on its GAPDH mRNA content. The results are expressed as fold change respect to the vehicle-treated cells. The values represent the mean ± SD of three different experiments, each performed with triplicate samples. *P < 0.05. n.s. = not significant.

The results obtained prompted us to determine whether the human CXCR4 gene may be a target of ligand-activated PPARγ. To this aim, transient transfection experiments were performed in MCF-7 cells using a luciferase reporter plasmid containing the human CXCR4 promoter region spanning from −2237 bp to +62 bp relative to the start of the transcription, named p-2300 (Figure 2A). BRL administration induced a significant reduction of CXCR4 promoter activity, which was reversed by the addition of GW, indicating that it was mediated by PPARγ activation (Figure 2A). The CXCR4 promoter region presents multiple transcription factor binding motifs, including c/EBP, Oct-1, NFkB and Sp1 that may represent potential PPARγ binding sequences [35, 45–47]. To evaluate which elements in the CXCR4 promoter can mediate the above described effects, CXCR4 promoter deleted constructs were tested in transient transfection experiments (Schematically reported in Figure 2A). By using p-2144 (−2144/+62) construct, the reduced luciferase activity upon BRL treatment was still present, whereas when we used the construct p-1507 (−1507/+62) the downregulatory effects were no longer noticeable (Figure 2A). This addresses that the region between −2144 and −1507 bp is required for the BRL-induced repression of CXCR4 promoter and may contain putative PPARγ responsive region(s). Our subsequent studies were directed to identify the putative sequence responsive to PPARγ within the promoter region of the *CXCR4* gene. Interestingly, nucleotide sequence analysis revealed that CXCR4 promoter contains the sequence AGGATAcAGATGA located at position −1761 upstream of the translation initiation codon, spanning from 136119895 bp to 136119907 bp on chromosome 2 (Figure 2B), that displays a high sequence homology with the canonical PPAR response elements (PPRE). We then compared our putative PPRE sequence with a consensus one generated using a PPRE collection from the literature [48] and visualized as a ‘sequence logo’. As shown in Figure 2C, we observed that the two motif profiles exhibited many similarities, particularly in the first hexad sequence bound to PPARγ, the nucleotides AGG located at position 1–3 as well as the nucleotide A located at position 6 are present in the putative PPRE sequence, suggesting the existence, within the CXCR4 promoter, of a novel PPRE-like region. To further investigate the functional importance of the identified PPRE sequence, we tested the hypothesis that PPARγ could effectively bind to it. To this aim, DNA affinity precipitation assay (DAPA) was performed in MCF-7 cells by using a biotinylated-double-stranded oligonucleotide containing the putative PPRE sequence (Figure 3A). Endogenous PPARγ was found to be associated with the putative consensus oligonucleotide following BRL treatment. Co-treatment with GW markedly decreased the BRL-induced DNA-binding complex demonstrating the direct involvement of PPARγ. A mutant oligonucleotide abolished PPARγ binding, indicating that the *in vitro* DNA-PPARγ binding is sequence-specific. Next, to assess whether the endogenous PPARγ, after BRL treatment, localizes to the native CXCR4-promoter, chromatin immunoprecipitation (ChIP) assay was performed by using primers flanking the PPRE sequence present in the CXCR4 promoter region. PPARγ occupancy of this region was significantly enhanced upon BRL treatment. This event was concomitant with the inhibition of RNA POL II recruitment onto the CXCR4 promoter (Figure 3B). Transcriptional control by PPARγ requires interaction with co-regulator complexes, either a coactivator for stimulation or a corepressor for inhibition of target gene expression [49–51]. To determine if the negative regulation of the CXCR4 transcriptional activity induced by BRL might be caused by the cooperative interaction between PPARγ and negative transcriptional regulators, we investigated the involvement of N-CoR and SMRT, which interact with and function as negative coregulators of PPARγ. Re-ChIP assay demonstrated a significant increase of PPARγ/SMRT complex occupancy of the PPRE containing region of CXCR4 promoter after BRL exposure. No interaction of N-CoR was observed under the same experimental conditions (Figure 3C). Finally, to better define the role of SMRT in the PPARγ-dependent modulation of the CXCR4 levels, RNA silencing technologies were used to knockdown the expression of endogenous SMRT in MCF-7 cells. SMRT expression was effectively silenced as revealed by real-time PCR analysis after 48 h of siRNA transfection (Figure 3D, upper panel). As expected, silencing of SMRT completely abrogated the down-regulation of CXCR4 mRNA levels induced by the activated PPARγ (Figure 3D, lower panel), highlighting a crucial role of SMRT corepressor in regulating CXCR4 expression upon BRL treatment. All these BRL-induced effects were reversed in presence of combined treatment with GW (Figure 3B–3D). Overall, these findings clearly demonstrated that ligand-activated PPARγ by binding to a newly identified PPRE motif within the CXCR4 promoter downregulates CXCR4 expression levels in human breast cancer cells.

### BRL inhibits motility in breast cancer cells

**Figure 4:**
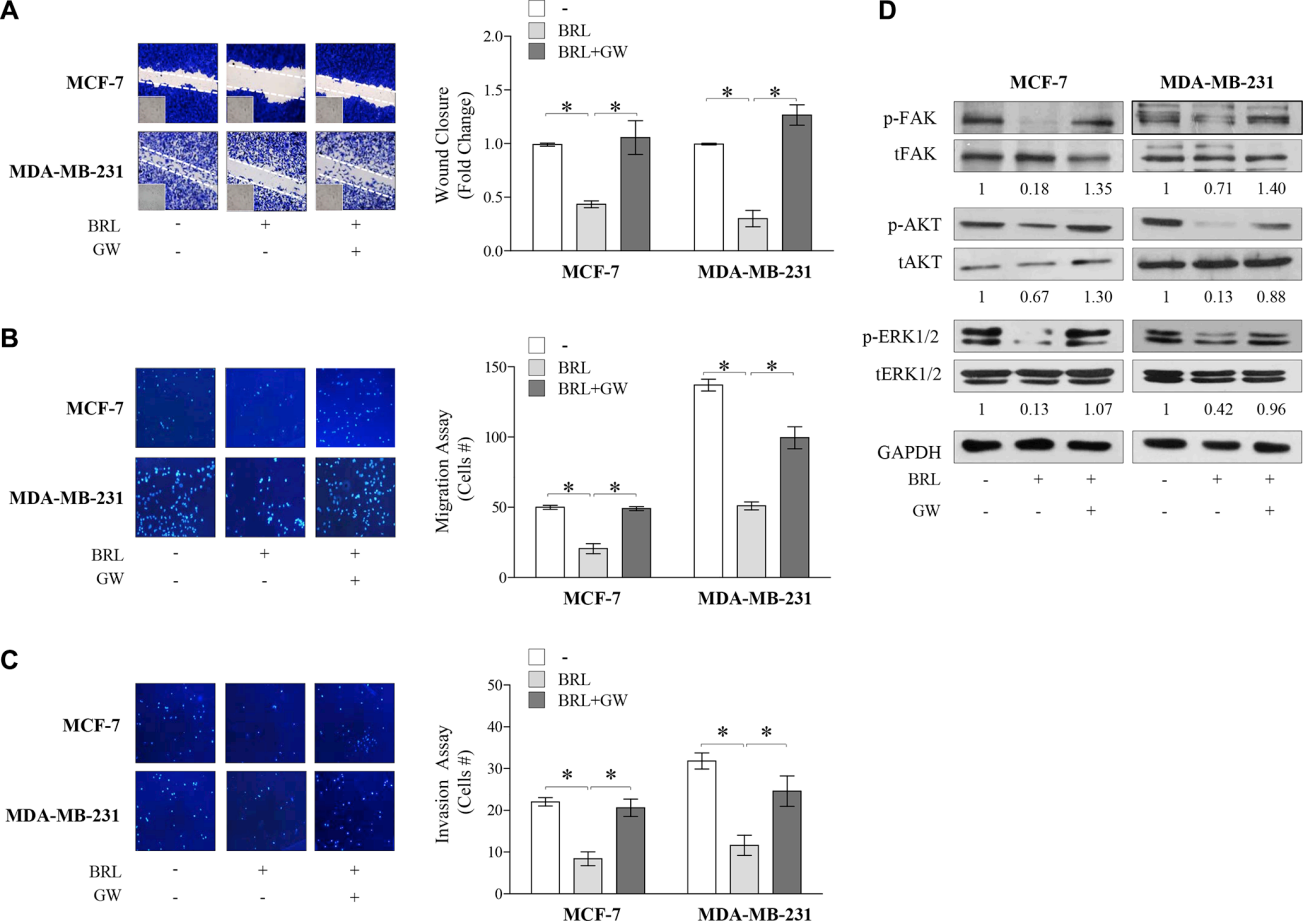
Effects of BRL on motility and invasion of MCF-7 and MDA-MB-231 breast cancer cells Wound-healing (**A**), transmigration (**B**) and invasion (**C**) assays in breast cancer cells treated with vehicle (−), BRL 10 μM with or without GW 10 μM for 24 h. Small squares: time 0. Histograms in A represent the mean ± SD of three separate experiments in which migrated cells were calculated by image analysis using Image J software and expressed as fold change compared to vehicle-treated cells. Migration and invasion were quantified by viewing five-separate fields/membrane (10**×**-magnification) and expressed as mean numbers of migrated cells. Data represent the mean ± SD of three-independent experiments, assayed in triplicate. *P < 0.05. (**D**) Immunoblots of phosphorylated levels (p) of FAK, AKT and ERK1/2 and total proteins from cells treated with vehicle (−), BRL 10 μM with or without GW 10 μM for 24 h. Numbers below the blots represent the average fold change between phosphorylated and total protein and GAPDH protein expression *vs* vehicle-treated cells. GAPDH, glyceraldehyde-3-phosphate dehydrogenase.

Given the largely documented role of SDF-1α/CXCR4 axis in modulating cancer cell migration [52–54], we next assessed the ability of PPARγ agonist to influence cell migration and invasion of both breast cancer cells. First, ELISA measurement in breast cancer cell media showed that SDF-1α levels were 171,6 ± 24,5 pg/mL and 143,35 ± 52,9 pg/mL in MCF7 and MDA-MB-231 cell-derived conditioned media (CM), respectively. Thus, we tested the capacity of cells to migrate in wound-healing scratch assays as well as to across uncoated membrane in transmigration assays and to invade an artificial basement membrane Matrigel in invasion assays upon treatment with BRL at 10 μM of concentration for 24 h (Figure 4A–4C). Our data clearly showed that BRL treatment significantly reduced motility and invasion in MCF7 and MDA-MB-231 cells, interfering with the autocrine effects of SDF-1α/CXCR4 system in these cells. These effects were abrogated when cells were exposed to GW co-treatment (Figure 4A–4C). Moreover, we observed, as expected, that ligand-activated PPARγ reduced breast cancer cell migration induced by SDF-1α (data not shown). We also tested the effects of ligand-activated PPARγ on CXCR4 downstream signaling pathways and we found decreased levels of phosphorylated FAK, AKT and ERK1/2 upon BRL treatment which was reversed in presence of GW, confirming that BRL reduces the CXCR4 signaling in a PPARγ-dependent manner in both breast cancer cell lines (Figure 4D). Moreover, we ascertained that the inhibited migratory capability mediated by BRL was not due to a decrease in cell viability, since when MCF-7 and MDA-MB-231 cells were incubated with 10 μM BRL for 24 h ∼90% of breast cancer cells were still viable (Supplementary Figure S2A).

### Ligand-activated PPARγ counteracts stroma-mediated breast cancer cell migration

**Figure 5:**
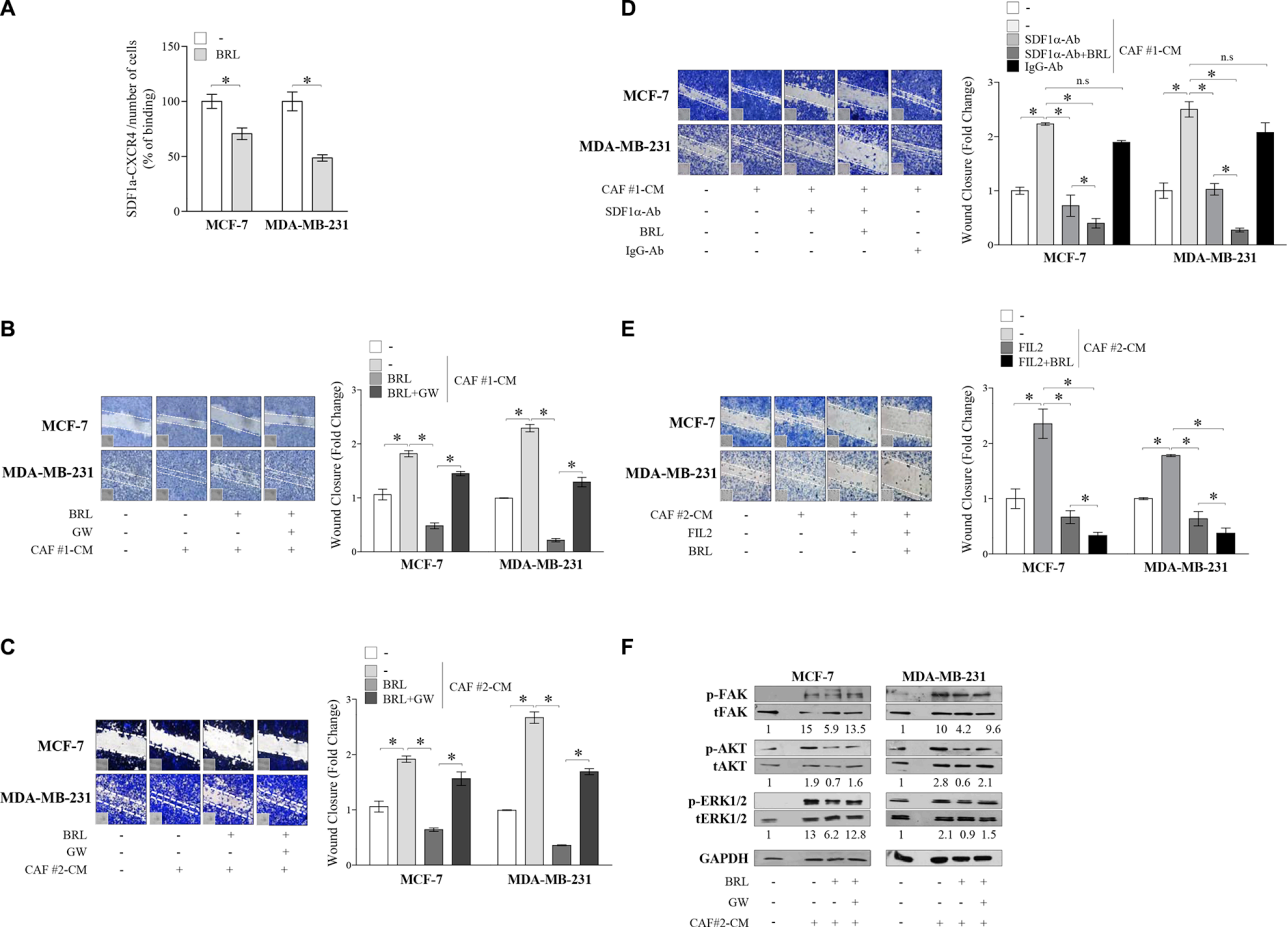
BRL antagonizes motility and signaling activation induced by cancer-associated fibroblasts -derived conditioned media in breast cancer cells (**A**) CAF-secreted SDF-1α ligand binding to breast cancer cells was analyzed by ELISA at 450 nm of absorbance (Abs) as described in Material and Methods. The results are expressed as percentage of optical density (OD) respect to vehicle-treated cells. The values represent the mean ± SD of three different experiments, each performed with triplicate samples. (**B** and **C**) Wound-healing assays in MCF-7 and in MDA-MB-231 cells treated with phenol-red and serum-free medium (−), conditioned media derived from cancer-associated fibroblasts (CAF-CM), BRL 10 μM with or without GW 10 μM for 24 h. Small squares, time 0. Histograms represent the mean ± SD of three separate experiments in which migrated cells were calculated by image analysis using Image J software and expressed as fold change compared to vehicle-treated cells. *P < 0.05. (**D**) Wound-healing assays in MCF-7 and in MDA-MB-231 cells treated with phenol-red and serum-free medium (−), CAF #1-CM and/or SDF-1α-depleted conditioned media (SDF-1α-Ab) with or without BRL 10 μM for 24 h. Conditioned media treated with a nonspecific IgG as a control (IgG-Ab). Small squares, time 0. Histograms represent the mean ± SD of three separate experiments in which migrated cells were calculated by image analysis using Image J software and expressed as fold change compared to (−) treated cells. *P < 0.05. (**E**) Wound-healing assays in MCF-7 and in MDA-MB-231 cells treated with phenol-red and serum-free medium (−), CAF #2-CM, FIL2 1 μM with or without BRL 10 μM for 24 h. Small squares, time 0. Histograms represent the mean ± SD of three separate experiments in which migrated cells were calculated by image analysis using Image J software and expressed as fold change compared to (−) treated cells. *P < 0.05. (**F**) Immunoblots of phosphorylated (p) FAK, AKT and ERK1/2 and total proteins from cells treated as in C. Numbers below the blots represent the average fold change between phosphorylated, total and GAPDH protein expression *vs* vehicle-treated cells. GAPDH, glyceraldehyde-3-phosphate dehydrogenase. CAFs: Cancer-associated fibroblasts; CM: Conditioned media.

There is increasing evidence that breast cancer behavior reflects an interconnection between the malignant epithelial compartment and the surrounding microenvironment. Cancer Associated Fibroblasts (CAFs) represent the most abundant stromal cell type populating the tumor microenvironment and play a pivotal role in the development and progression of breast cancer via production of hormones, extracellular matrix remodeling enzymes and cytokines such as SDF-1α [4]. To investigate the role of activated PPARγ in the context of heterotypic signaling working in tumor-stroma interactions, we examined the ability of BRL to reduce CAF-induced effects through CXCR4 axis inhibition in breast cancer cells. To this aim, two different types of CAFs, named CAF #1 and CAF #2, isolated from biopsies of primary breast tumors, were used in co-culture systems. First, MCF-7 and MDAMB-231 cells were pretreated with BRL 10 μM for 24 h and then incubated with CAF-derived CM to assess stromal SDF-1α ligand binding to breast cancer cells. In line with BRL-induced CXCR4 downregulation, we observed a significantly decreased SDF-1α/CXCR4 binding in cells pretreated with BRL compared to vehicle-treated cells (Figure 5A). Accordingly, treatment with BRL attenuated migration-promoting activities of CM from CAF #1 and CAF #2 (Figure 5B and 5C). SDF-1α was then immunodepleted from CAF-derived CM by a specific antibody, and resulting media were tested in cells treated with BRL for the ability to reduce migration of breast cancer cells. As expected, SDF-1α-depletion (CAF-CM + SDF-1α-Ab) significantly reduced the migratory effects of CAF-CM, particularly in the presence of BRL treatment (Figure 5D). CM treated with a nonspecific rabbit IgG had no effects, suggesting the specificity of SDF-1α antibody. In addition, as shown in Figure 5E, BRL in combination with the CXCR4 antagonist FIL2, a newly benzohydrazide compound synthesized in our laboratory [13], strongly decreased cell motility induced by CAF-CM. Moreover, we demonstrated that BRL was also able to counteract the increased activation of FAK, AKT and MAPK signaling pathways induced by CM from CAFs in both breast cancer cells (Figure 5F). The PPARγ antagonist GW abolished the effects of BRL on migratory promoting activities induced by CAF-CM (Figure 5B, 5C and 5F).

### BRL affects phenotypic characteristics of CAFs

**Figure 6:**
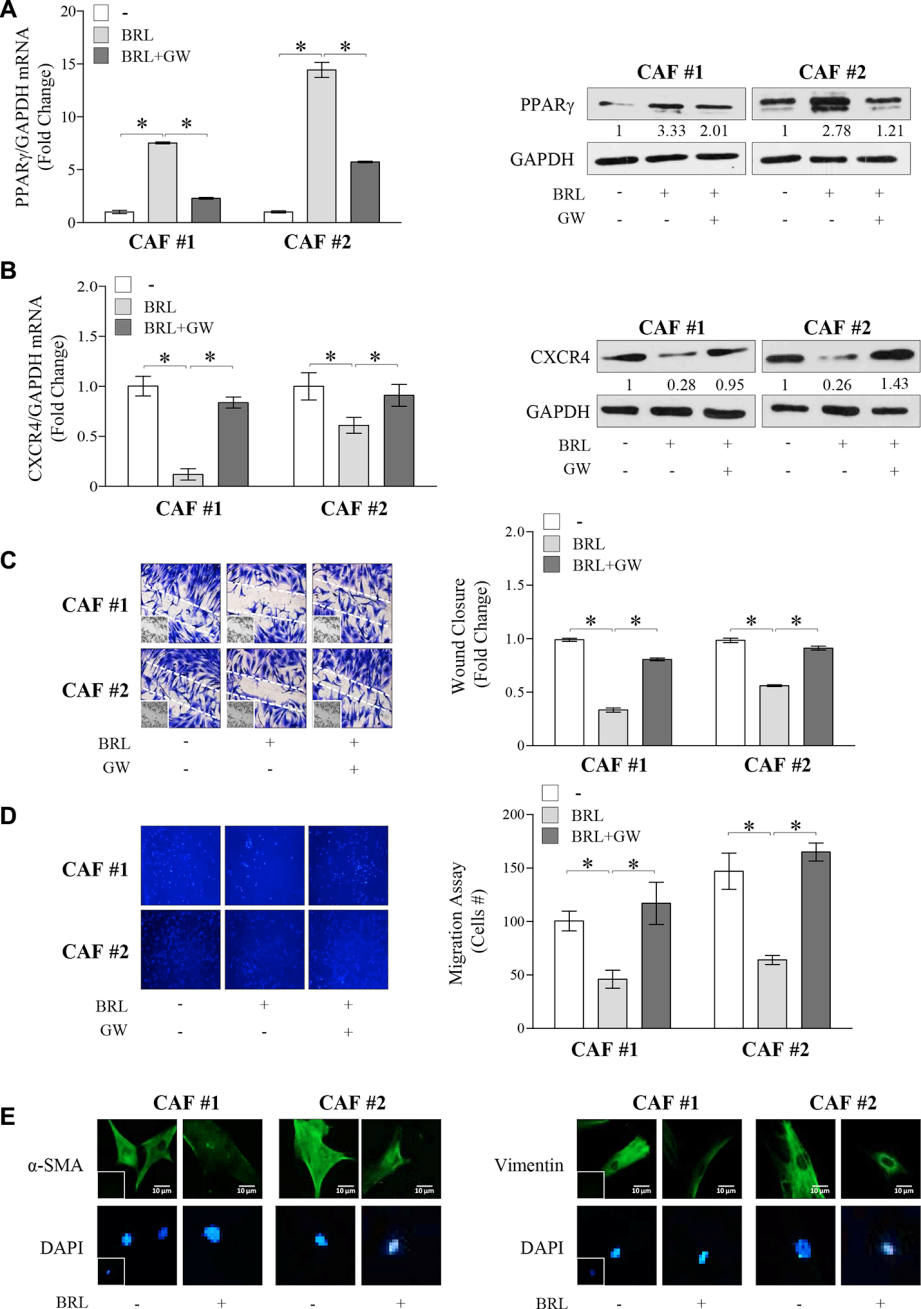
Effects of BRL on CAF phenotype (**A**) Real-time RT-PCR (*left panel*) and immunoblots (*right panel*) of PPARγ in Cancer-Associated Fibroblasts (CAFs) treated with vehicle (−), BRL 10 μM with or without GW 10 μM for 12 h and 24 h, respectively. Each sample was normalized on its GAPDH mRNA content. The results are expressed as fold change respect to vehicle-treated cells. The values represent the mean ± SD of three different experiments, each performed with triplicate samples. GAPDH was used as loading control. Numbers below the blots represent the average fold change between PPARγ and GAPDH protein expression *vs* vehicle-treated cells. (**B**) Real-time RT-PCR (*left panel*) and immunoblots (*right panel*) of CXCR4 in CAFs treated with vehicle (−), BRL 10 *μ*M with or without GW 10 μM for 12 h and 24 h, respectively. Each sample was normalized on its GAPDH mRNA content. The results are expressed as fold change respect to vehicle-treated cells. The values represent the mean ± SD of three different experiments, each performed with triplicate samples. GAPDH was used as loading control. Numbers below the blots represent the average fold change between CXCR4 and GAPDH protein expression *vs* vehicle-treated cells. *P < 0.05. GAPDH, glyceraldehyde-3-phosphate dehydrogenase. Wound-healing (**C**), transmigration (**D**) assays in CAFs treated with vehicle (−), BRL 10 μM with or without GW 10 μM for 24 h. Small squares: time 0. Histograms in C represent the mean ± SD of three separate experiments in which migrated cells were evaluated with ImageJ and expressed as fold change. Migration in D was quantified by viewing five-separate fields/membrane (10**×**-magnification) and expressed as mean numbers of migrated cells. Data represent the mean ± SD of three-independent experiments, assayed in triplicate. (**E**) Immunofluorescence of α-SMA and Vimentin in CAFs treated with vehicle (−) or BRL 10 μM for 24 h. Small squares, negative controls. 4,6-Diamidino-2-phenylindole (DAPI) was used for the determination of the nuclei. Scale bar, 10 μm.

As a final step of this study, we wondered whether PPARγ ligands by influencing CXCR4 expression may also impact biological features of CAFs. As previously reported [55], we found that CAFs showed a detectable mRNA and protein levels of PPARγ which was significantly increased upon 10 μM BRL exposure and reversed by GW co-treatment (Figure 6A). In addition, we observed that exposure to BRL reduced, in a PPARγ-dependent manner, CXCR4 expression evaluated at both mRNA and protein levels (Figure 6B). As a consequence, BRL treatment reduced CAF motility assessed by wound healing and trans-migration assays (Figure 6C and 6D). The ability of GW to completely abrogate this effect addressed a direct involvement of PPARγ. It was observed that incubation with 10 μM BRL for 24 h did not affect cell viability of CAFs (Supplementary Figure S2B), while interestingly BRL elicited a dramatic alteration in the shape of CAFs *in vitro* (data not shown), accompanied by a reduced expression of α-SMA and vimentin in both types of CAFs (Figure 6E). Taken together our results indicate that CAFs exposed to BRL acquired a phenotype characterized by an altered morphology, a decreased expression of CXCR4 and inhibited migratory capabilities, all features that may negatively impact breast tumor progression.

## DISCUSSION

Several reports have demonstrated that chemokines and their receptors play critical roles in the development and progression of cancer by controlling cell survival, proliferation, invasion and metastasis. The complex interaction between chemokines and their receptors regulates the response of target cells, acting directly on tumor or host cells and giving rise to a diversity of effects that shape the malignant phenotype in the tumor microenvironment [5–7, 16]. Out of all the known chemokine receptors, breast cancer cells specifically express active CXCR4, highly associated with metastatic potential of human breast cancer [56–58]. Therefore, novel drugs capable of downregulating the CXCR4 axis may demonstrate potential for breast cancer treatment.

Here, we identified, for the first time, CXCR4 as a novel target gene of PPARγ and demonstrated that its expression is negatively modulated by the ligand-activated PPARγ. Indeed, in breast cancer cells, CXCR4 expression is downregulated by administration of the TDZ drug BRL as evidenced by reduction of its mRNA and protein levels. Accordingly, previous observations have reported that PPARγ ligands downregulate CXCR4 expression in colon, lung and prostate cancer cells [40–42, 59], however, the mechanism by which PPARγ may regulate CXCR4 expression remain largely unknown. Thus, we focused on the molecular mechanism by which PPARγ mediates the inhibition of CXCR4 expression in breast tumor cells. We have demonstrated by functional studies that activated PPARγ decreased CXCR4 promoter activity and that the region between −2144 bp and −1507 bp was essential for the downregulation exerted by BRL. Specifically, the nucleotide sequence analysis of this region revealed a putative PPAR response element (PPRE-like: 5-AGGATAcAGATGA-3) located between −1761 bp and −1748 bp upstream of the CXCR4 gene translation initiation codon, which corresponds to the sequence spanning from 136119907 to 136119895 bp on the long (q) arm of chromosome 2 at position 2q21. It is well known that the consensus sequence of the PPRE is composed of 2 hexad sequences (AGGTCA) directionally aligned and separated by a single nucleotide spacer (DR-1, direct repeat), and PPARγ has been shown to occupy the 5′ half-site of the DR-1 element, with RXR occupying the 3′ half-site. None of the endogenous PPREs thus far identified possess the canonical consensus sequence, rather, the majority of actual PPREs represent degenerate sequences [60]. Interestingly, comparing the putative PPRE motif to a sequence logo generated using internet-based software tools from a set of PPREs found in the promoters of several PPARγ-responsive genes [49], we predict the existence of a novel PPRE-like region within the CXCR4 promoter. This PPRE is functional, as demonstrated by transactivation studies, and capable to efficiently bind to PPARγ in a ligand-dependent manner. Furthermore, the *in vivo* interaction between PPARγ and the CXCR4 promoter is supported by ChIP analysis showing that PPARγ occupancy of the CXCR4-PPRE containing promoter region was concomitant with a decrease in RNA Polymerase II recruitment, consistent with the suppressed CXCR4 transcriptional activity. It has been reported that the negative transcriptional control by PPARγ occurs through its recruitment on the own binding site within the promoter of target genes in association with negative transcriptional corepressors, such as SMRT and NCoR [61]. Re-ChIP assays in cells treated with BRL showed an increased recruitment of the negative transcriptional regulator SMRT onto the PPRE site within the CXCR4 promoter leading to inhibition of gene transcription. The direct involvement of SMRT in the CXCR4 promoter responsiveness to the BRL has been demonstrated after RNAi-mediated inhibition of this corepressor in breast cancer cells. Collectively, our study by identifying a PPRE-like sequence within CXCR4 promoter provides the molecular mechanism by which activated PPARγ downregulates CXCR4 expression, thus contributing to explain the negative influence of BRL on breast cancer cell motility and invasion, interfering with the autocrine effects of SDF-1α/CXCR4 system in these cells. The molecular mechanisms by which PPARγ exerts its anti-invasive functions have not yet been defined, although PPAR*γ* agonists have been shown to regulate matrix metallopeptidases (MMPs), tissue inhibitors of MMPs and E-cadherin expression levels as well as to interfere with estrogen receptor, STAT5B, NF-kB and tumor growth factor-*β* signalling cascades [62–64]. Many current lines of evidences highlight the existence of a crosstalk between PPARγ activity and death signaling pathways leading to anti-proliferative effects, cell-cycle arrest, apoptosis and autophagy in human breast cancer cells, however, these effects occur at high doses and/or after long-term treatment [34, 36–37, 65–66]. In the present study, we observed that BRL at 10 μM of concentration for 24 h did not decrease cell viability but it was able to inhibit, in a PPARγ-dependent manner, migration and invasion of breast cancer cells. It is now well established that the tumor progression is highly dependent on interactions between malignant cells and stromal cells within tumor microenvironment [67–68]. Reactive stroma is composed of several heterotypic cells, among which CAFs represent one of the most abundant cell types of different carcinomas including breast cancer. CAFs are activated fibroblasts which communicate among themselves as well as with cancer cells through a complex network able to support tumorigenesis, angiogenesis, and metastasis [57, 69]. Indeed, CAFs have higher expression of SDF-1α than those of normal breast tissue, and through this paracrine signaling, CXCR4 may promote local tumor cell proliferation, motility and invasion [19]. Our findings demonstrated that BRL inhibited CAF-induced effects on cell motility and downstream signaling activation in different breast cancer cellular backgrounds. Administration of the PPARγ antagonist GW9662 completely abrogated the effect of BRL on the motile and invasive behavior, highlighting a role for PPARγ activation in interfering with the paracrine effects of SDF-1α/CXCR4 axis in malignant breast epithelial cells. Moreover, the establishment of the autocrine signaling loop mediated by SDF-1α in CAFs acts to maintain their tumor-promoting phenotype [20]. Fibroblasts in the tumor stroma present a very heterogeneous cell population, reflected both by the variable morphological appearance and variable expression of CAF-markers within the individual tumor. Indeed, the activated fibroblasts, which are characterized by enhanced contractile property, display an increased expression of α-SMA that has been implicated in contractile activity of fibroblasts [70]. Interestingly, our data showed that CAFs exposed to the treatment with the PPARγ agonist BRL acquired a phenotype characterized by a decreased expression of α-SMA/vimentin and CXCR4 together with a reduced migratory capability, all features that may negatively impact breast tumor progression.

In conclusion, our data highlight a novel role for PPARγ in controlling breast cancer progression and in affecting CAF behavior. Given the key role played by the crosstalk between cancer cells and CAFs in the progression of cancer, strategies aimed to specifically target both components may represent an important approach to improve patient outcome. Together with low toxicity profiles of PPARγ ligands, our findings may offer promising insights into future treatments at least in more aggressive and/or drug-resistant breast tumor phenotypes.

## MATERIALS AND METHODS

### Reagents

Rosiglitazone (BRL49653, BRL) was obtained from Alexis (San Diego, CA), GW9662 (GW) and 15-deoxy-Delta 12,14-prostaglandin J2 (PGJ2) from Sigma Aldrich (Milan, Italy) and Stromal-cell Derived Factor-1alpha (SDF-1α) from Prospec (Rome, Italy). 2-(5-Bromo-1H-indol-1-yl)-N′-(pyrazin-2-yl) benzohydrazide (FIL2) was kindly provided by Dr. Grande.

### Plasmids

The human CXCR4 gene promoter constructs (p-2300, p-2144, p-1507) were a gift from Prof. M. Z. Ratajczak (Stem Cell Institute at James Graham Brown Cancer Center, University of Louisville, Louisville, KY).

### Cell culture

Human ERα-positive MCF-7 and the triple-negative (ER-, PR- and Her2-negative) MDA-MB-231 breast cancer epithelial cells were acquired from American Type Culture Collection where they were authenticated, stored according to supplier's instructions, and used within 4 months after frozen aliquots recovery. Every 4 months, cells were authenticated by single tandem repeat analysis at our Sequencing Core; morphology, doubling times, estrogen sensitivity, and mycoplasma negativity were tested (MycoAlert, Lonza). MCF-7 cells were cultured in DMEM (Life Technologies, Carlsbad, CA, USA) supplemented with 10% fetal bovine serum (FBS) (Life Technologies), 1 mg/ml penicillin-streptomycin (Life Technologies) and 0.01 mg/ml insulin (Sigma Aldrich) at 37°C with 5% CO_2_ air. MDA- MB-231 cells were cultured in DMEM/F-12 plus glutamax (Life Technologies) containing 10% FBS and 1 mg/ml penicillin-streptomycin. MCF-10A non tumorigenic breast epithelial cells were grown in DMEM-F12 plus glutamax containing 5% horse serum (HS) (Life Technologies), 1 mg/ml penicillin–streptomycin, 0.5 mg/ml hydrocortisone (Sigma Aldrich), and 10 mg/ml insulin. For experimental purposes, cells were grown in phenol red-free media containing 5% charcoal-treated FBS (CT-FBS) for 24 h and then treated as described.

### Cancer associated fibroblast (CAF) isolation

Human breast cancer specimens were collected in 2013–2014 from primary tumors of patients who signed informed consent in accordance with approved Human Subject's guidelines at Annunziata Hospital (Cosenza, Italy), following the procedures previously described [71]. Briefly, small pieces of fresh tumor excision were digested (500 IU collagenase in Hank's balanced salt solution; Sigma Aldrich; 37°C for 2 h). After differential centrifugation (90 g for 2 min), the supernatant containing CAFs was centrifuged (500 g for 8 min), resuspended, and cultured in MEDIUM 199 (Life Technologies)/F-12 (Sigma Aldrich) (1:1) supplemented with 15% FBS and antibiotics. The fibroblastic nature of the isolated cells was confirmed by microscopic determination of morphology, and characterization by α-SMA, vimentin. CAFs between 4 and 10 passages were used.

### Conditioned medium systems

Cells were incubated with regular full media (48 h). Conditioned media (CM) were collected, centrifuged to remove cellular debris, and used in respective experiments.

### Cell viability assay

Cell viability was determined with the 3-(4,5-dimethylthiazol-2-yl)-2,5-diphenyltetrazolium (MTT) assay. Cells (40,000 cells/well) were grown in 24-well plates and exposed to treatments as indicated. MTT (2 mg/ml, Sigma Aldrich) was added to each well, and the plates were incubated for 2 h at 37°C followed by medium removal and solubilization in 500 μl DMSO (Sigma Aldrich). The absorbance was measured at 570 nm.

### Immunoblot analysis

Cells were treated as indicated before lysis for total protein extraction [38]. Equal amounts of cell extract proteins were resolved on 8–11% SDS-polyacrylamide gels, transferred to nitrocellulose membranes, and probed with anti-CXCR4 (BD Biosciences, San Jose, CA, USA), -PPARγ, -pFAK (Tyr^576/577^), -FAK, -pAKT (Ser^473^), -AKT, -GAPDH (Santa Cruz Biotechnology, Santa Cruz, CA, USA), and -pERK 1/2 (Thy^202^/Tyr^204^), -ERK 1/2 (Cell Signalling Technology, Danvers, MA, USA) antibodies. The antigen-antibody complex was detected as previously described [38].

### RT-PCR/qRT-PCR

Analysis of gene expression was performed using qRT-PCR. Total cellular RNA was extracted using TRIZOL reagent (Life Technologies) as suggested by the manufacturer. The purity and integrity were checked spectroscopically and by gel electrophoresis before carrying out the analytical procedures. Two micrograms of total RNA were reverse transcribed in a final volume of 20 μL using a RETROscript kit (Applied Biosystems, Monza, Italy) as suggested by the manufacturer. cDNA was diluted 1:3 in nuclease-free water and 5 μl were analyzed in triplicates by qRT-PCR in a iCycler iQ Detection System (Bio-Rad, Milan, Italy) as previously described [65]. Negative control contained water instead of first strand cDNA was used. Each sample was normalized on its GAPDH mRNA content. The primers set used were:

5′-AATCTTCCTGCCCACCATCT-3′CCACCT-3′ *(CXCR4-reverse)*, 5′-TTACCCGCAAAAGACAAGT-3′ *(SDF-1α forward)*, 5′-AGGCAATCACAAAACCCAGT-3′ *(SDF 1α reverse)*, 5′-CACCCGGCAGTATCATGAGA-3′ *(SMRT-forward)*, 5′-CGAGCGTGATTCCTCCTCTT-3′ *(SMRT-reverse)*, 5′-GGCTTCATGACAAGGGAGTTTC-3′ *(PPARγ-forward)*, 5′-AACTCAAACTTGGGCTCCATAA AG −3′*(PPARγ-reverse)*, 5′-CCCACTCCTCCACCTTTG AC-3′ *(GAPDH-forward)*, 5′-TGTTGCTGTAGCCAAATT CGTT-3′ *(GAPDH-reverse)*.

### Transient transfection assay

Cells were transiently transfected using X-TREME reagent (Roche, Indianapolis, IN, USA) with CXCR4 promoter-luciferase constructs. After transfection, cells were treated as indicated. Luciferase activity was assayed as previously described [65].

### Immunofluorescence

Cells were fixed with 4% paraformaldehyde, permeabilized with PBS 0.2% Triton X-100 followed by blocking with 5% bovine serum albumin, and incubated with anti-CXCR4 (BD Biosciences), anti-vimentin (Santa Cruz Biotechnology) and anti-α-SMA (Sigma Aldrich) antibodies and with fluorescein isothiocyanate-conjugated secondary antibodies. IgG primary antibody was used as negative control. 4′,6-Diamidino-2-phenylindole (DAPI; Sigma Aldrich) staining was used for nuclei detection. Fluorescence was photographed with OLYMPUS BX51 microscope, 100**×** objective.

### Chromatin immunoprecipitation assay

Cells were treated with BRL for 1 h and then DNA/protein complexes were extracted as described [72]. The immuno-cleared chromatin was precipitated with specific anti- PPARγ and anti-Polymerase II (POLII) (Santa Cruz Biotechnology) antibodies. The anti-PPARγ immunoprecipitated samples were re-immunoprecipitated (Re-ChIP) with an anti-NCoR and anti-SMRT antibodies (Santa Cruz Biotechnology). A 5 ml of each sample and input were used for real-time-PCR. The primers flanking the PPRE sequence present in the CXCR4 promoter region were the following: 5′-CCACTACCAGGCTTTGTGAA-3′ and 5′-CGTAATGCAAGGCCTGTGAG-3′. Final results were calculated using the ΔΔCt method using input Ct values instead of the GAPDH. The basal sample was used as calibrator.

### DNA affinity precipitation assay

DNA affinity precipitation assay was performed as previously described [73]. The DNA motif probes were prepared by annealing a biotinylated sense oligonucleotide (for CXCR4-PPRE, 5′-[Bio]-TTATAAAGGATACAGATGA AGAGATACG-3′; for CXCR4-mutated PPRE, 5′-[Bio]-TTATAACTTATACAGA CTCAGAGATACG-3′ with the respective unbiotinylated complementary oligonucleotide (for CXCR4-PPRE, 5′-CGTATCTCTTCATCTGTATCCTTTATAA-3′; for CXCR4- mutated PPRE, 5′-CGTATCTCTGAGTCTG TATAAGTTATAA-3′.

### RNA silencing

Cells were transfected with RNA duplex of stealth siRNA targeted for the human PPARγ mRNA sequence 5′-AGA AUA AUA AGG UGG AGA UGC AGG C-3′ (Life Technologies), human SMRT mRNA sequence (Ambion, ID:s74031) or with a control siRNA used as a control for non-sequence-specific effects to a final concentration of 100 nM using Lipofectamine 2000 (Life Technologies) as recommended by the manufacturer. After 6 h the transfection medium was changed 5% CT-FBS for 48 h and then the cells were exposed to treatments.

### Wound-healing assays

For the measurement of cell migration during wound healing, confluent cell cultures were incubated in phenol-red and serum-free medium for 24 h before the beginning of the experiment. Cell monolayers were then scraped, washed to remove debris and treated as indicated in the respective experiments. Wound closure was monitored over 24 h. Cells were then fixed, stained with Comassie Brillant Blue and photographed after wounding under phase contrast microscopy at 10**×** magnification. The rate of wound healing was quantified from the images using Image J and standard deviations along with associated *P* values for the biological replicates were determined by using GraphPad-Prism5 software (GraphPad Inc., San Diego, CA). Pictures represent one of three-independent experiments.

### Transmigration assays

Cells under the various experimental conditions were placed in upper compartments of Boyden-chambers (8 μm-membranes, Corning). Bottom well contained regular-growth media. After 24 h, migrated cells were fixed and stained with DAPI. Migration was quantified by viewing five-separate fields/membrane (OLYMPUS-BX51 microscope, 10**×**-magnification) and expressed as mean numbers of migrated cells. Data represent three-independent experiments, assayed in triplicate.

### Invasion assays

Matrigel-based invasion assay was performed in Boyden-chambers (8 μm-membranes) coated with Matrigel (BD Bioscences, 0.4 μg/ml), as described [74]. After 24 h, invaded cells were quantified as reported for transmigration assays.

### CXCL12/SDF-1α ELISA

SDF-1α was measured in CM from MCF-7 and MDA-MB-231 cells using a commercially available ELISA Kit in accordance with the instructions by the manufacturer (Human CXCL12/SDF-1 alpha Quantikine ELISA Kit, R&D Systems, Inc. Minneapolis, USA).

For binding assay, breast cancer cells were untreated (-) or treated with BRL 10 μM in phenol red-free media containing 5% CT-FBS for 24 h. Then, cells were harvested with versene reagent, washed twice in PBS and 10^3^ cells/well were incubated with CAF-CM in a final volume of 100 μl binding buffer (50 mM HEPES, pH 7.4, 1 mM CaCl2, 150 mM NaCl, 5 mM MgCl2, 5% bovine serum albumin). Samples were incubated for 60 min at 4°C with rotation. After incubation, cells were centrifuged and washed twice with 300 μl wash buffer (50 mM HEPES, pH 7.4, 1 mM CaCl2, 500 mM NaCl, 5 mM MgCl2) and freezed to −20°C and thawed to room temperature 3 times and then centrifuged at 1500×g for 10 minutes at 2 − 8°C to remove cellular debris. The supernatants were collected for assaying human SDF-1α levels (R&D Systems). The optical density of each well was determined using a microplate reader at 450 nm (Bio-Rad Model 3550 microplate reader, Richmond, CA) and normalized for cell number. At least three independent experiments were performed.

### SDF-1α -immunodepleted conditioned media

Protein G-agarose beads were incubated with anti-SDF-1α (Cell Signalling Technology) or IgG antibodies. Antibody-beads complexes were incubated with CAF-derived CM and centrifuged. SDF-1α immunodepletion was verified by ELISA.

### Statistical analysis

Each datum point represents the mean ± SD of three different experiments. Experimental data were analyzed for statistical significance by one-way ANOVA test using the GraphPad Prism5 software program. *P < 0.05 was considered as statistically significant.

## SUPPLEMENTARY MATERIALS FIGURES



## Abbreviations

PPARγ: Peroxisome Proliferator-Activated Receptor gamma
PPRE: Peroxisome Proliferator-Activated Receptor Response Element
SDF-1 a: Stromal Derived-Factor-1a
CAF: Cancer-Associated Fibroblast
SMRT: Silencing Mediator of Retinoid and Thyroid hormone receptor
