# Supplementary material for: Ligand-activated PPARγ downregulates CXCR4 gene expression through a novel identified PPAR response element and inhibits breast cancer progression
Source: Oncotarget. 2016 Aug 18;7(40):65109–24. doi: 10.18632/oncotarget.11371 (PMC5323141; doi:10.18632/oncotarget.11371)
Supplement: Supplementary file 1 [file oncotarget-07-65109-s001.pdf]

Ligand-activated PPARγ downregulates CXCR4 gene expression through a novel identified PPAR response element and inhibits breast cancer progression

Supplementary Materials

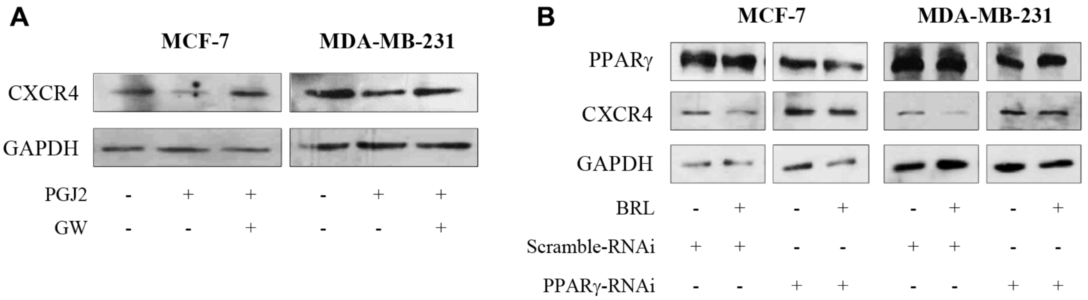

Supplementary Figure S1: (**A**) Immunoblots of CXCR4 protein expression in MCF-7 and MDA-MB-231 cells treated with vehicle (−), PGJ2 at 10 µM with or without GW 10 µM for 24 h. GAPDH was used as loading control. (**B**) Immunoblots of CXCR4 protein expression in MCF-7 and MDA-MB-231 cells transfected with scramble RNA interference (RNAi) or with PPARγ RNAi as reported in Materials and Methods Section and treated with vehicle (−) or with BRL 10 µM for 24 h. GAPDH was used as loading control.

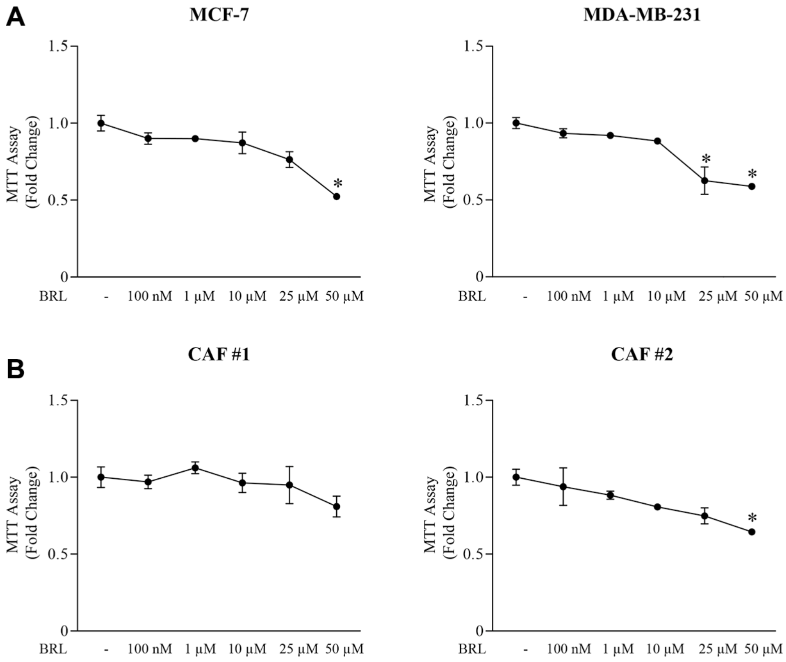

Supplementary Figure S2: Cell viability was determined by 3-(4,5-dimethylthiazol-2-yl)-2,5-diphenyltetrazolium (MTT) assays in MCF-7 and MDA-MB-231 breast cancer cells (A) and in Cancer-Associated Fibroblasts (CAF) (B) treated with vehicle (−) or with increasing concentrations (100 nM, 1, 10, 25, 50 µM) of BRL for 24 h. The results are expressed as fold change respect to vehicle-treated cells. The values represent the mean ± SD of three different experiments, each performed with triplicate samples. *$P < 0.05$ vs vehicle-treated cells.
